# Serum sphingolipids level as a novel potential marker for early detection of human myocardial ischaemic injury

**DOI:** 10.3389/fphys.2013.00130

**Published:** 2013-06-13

**Authors:** Emmanuel E. Egom, Mamas A. Mamas, Sanoj Chacko, Sally E. Stringer, Valentine Charlton-Menys, Magdi El-Omar, Debora Chirico, Bernard Clarke, Ludwig Neyses, J. Kennedy Cruickshank, Ming Lei, Farzin Fath-Ordoubadi

**Affiliations:** ^1^Department of Physiology and Biophysics, Faculty of Medicine, Dalhousie UniversityHalifax, NS, Canada; ^2^Faculty of Medicine and Human Sciences, Institute of Cardiovascular Sciences, University of ManchesterManchester, UK; ^3^Biomedical Research Centre, Central Manchester NHS Foundation TrustManchester, UK; ^4^Manchester Royal Infirmary, Manchester Heart CentreManchester, UK

**Keywords:** sphingolipids, sphingosine 1-phosphate, ischaemia

## Abstract

**Background:** Ventricular tachyarrhythmias are the most common and often the first manifestation of coronary heart disease and lead to sudden cardiac death (SCD). Early detection/identification of acute myocardial ischaemic injury at risk for malignant ventricular arrhythmias in patients remains an unmet medical need. In the present study, we examined the sphingolipids level after transient cardiac ischaemia following temporary coronary artery occlusion during percutaneous coronary intervention (PCI) in patients and determined the role of sphingolipids level as a novel marker for early detection of human myocardial ischaemic injury.

**Methods and Results:** Venous samples were collected from either the coronary sinus (*n* = 7) or femoral vein (*n* = 24) from 31 patients aged 40–73 years-old at 1, 5 min, and 12 h, following elective PCI. Plasma sphingolipids levels were assessed by HPLC. At 1 min coronary sinus levels of sphingosine 1-phosphate (S1P), sphingosine (SPH), and sphinganine (SA) were increased by 314, 115, and 614%, respectively (*n* = 7), while peripheral blood levels increased by 79, 68, and 272% (*n* = 24). By 5 min, coronary sinus S1P and SPH levels increased further (720%, 117%), as did peripheral levels of S1P alone (792%). Where troponin T was detectable at 12 h (10 of 31), a strong correlation was found with peak S1P (*R*^2^ = 0.818; *P* < 0.0001).

**Conclusion:** For the first time, we demonstrate the behavior of plasma sphingolipids following transient cardiac ischaemia in humans. The observation supports the important role of sphingolipids level as a potential novel marker of transient or prolonged myocardial ischaemia.

## Introduction

Despite recent advances in preventing sudden cardiac death (SCD) due to cardiac arrhythmia, its incidence in the population at large has remained unacceptably high. It is responsible for 50% of the mortality from cardiovascular disease in the developed countries and accounts for 300,000 to 400,000 deaths every year in the United States. About 80% of SCDs are caused by ventricular tachyarrhythmias that often occur without warning, leading to death within minutes in patients who do not receive prompt medical attention. It is the most common and often the first manifestation of coronary heart disease. Early detection/identification of acute myocardial ischaemia in patients at risk for lethal ventricular arrhythmias remains an unmet medical need. At *in vivo*, acute myocardial ischaemia is associated with dramatic electrophysiological alterations that may lead to malignant ventricular arrhythmias which occur within minutes of cessation of coronary flow and are rapidly reversible with reperfusion. This suggests that subtle and reversible biochemical and/or ionic alterations within or near the sarcolemma of myocardium during the early stage of acute ischaemic injury may contribute to the electrophysiological instability.

Sphingolipids are biologically active lipids (Alewijnse and Peters, 2008), whose serum sphingosine (SPH) levels were found to be elevated in animal models of myocardial infarction (MI; Zhang et al., 2001; Thielmann et al., 2002) and are thought to have an important cardioprotective role during the ischemic insult (Karliner et al., 2001). Sphingosine 1-phosphate (S1P) has been shown to be an important mediator of ischemic pre- and post-conditioning in both pharmacological and knockout animal studies (Karliner, 2009), with SIP receptors being expressed in the myocardium, endothelium, and platelets (Karliner, 2009). Deutschman et al. (2003) reported that sphingolipid levels are elevated in patients with coronary artery disease (CAD) and that S1P had a greater predictive value in detecting CAD, than traditional risk factors. It is not clear whether sphingolipids are markers of the inflammatory process associated with atherosclerotic CAD and/or are markers of cardiac ischaemia associated with flow obstructive coronary artery lesions. During coronary occlusion and subsequent reperfusion such as occurs during treatment of MI with percutaneous coronary intervention (PCI), reactive oxygen species (ROS) are formed that mediate ischaemia-reperfusion injury based on oxidative stress (Nikolic-Heitzler et al., 2006). ROS regulate S1P levels through changes in the function of sphingosine kinase, the final rate limiting step in S1P synthesis (Maceyka et al., 2007). Using samples collected from humans before and after balloon occlusion of coronary arteries during PCI we have for the first time investigated whether sphingolipids are elevated during brief periods of coronary occlusion and therefore transient cardiac ischaemia, hence providing novel insight into pathophysiological mechanisms that occur during ischaemia reperfusion injury and determined whether change of their level can be a novel marker for early detection of human myocardial ischemic injury. To understand whether oxidative stress during this transient ischaemia may potentially account for changes in sphingolipids level we also evaluated changes in oxidized LDL (Ox-LDL) used here as an oxidative stress biomarker.

## Methods

### Study protocol

This study complies with the Declaration of Helsinki, was approved by the North West 8 Research Ethics Committee of Greater Manchester East and all patients gave written informed consent before entry. Ethical approval was obtained from the National Research Ethics committee via the NRES committee-North West Greater Manchester Central, REC reference 07/H1008/162. Blood samples were obtained from 31 patients aged 40 to 73 years-old undergoing elective PCI to native coronary arteries at the Manchester Heart Centre, Manchester, UK. Procedures were performed via the femoral artery through standard 6Fr sheaths and peripheral venous samples were collected through a 6Fr femoral venous sheath. Coronary sinus sampling was performed using a 6Fr Amplatz Left-1 catheter (AL-1) during PCI. Control venous blood samples were obtained either from the coronary sinus (7 patients) or via the femoral venous sheath (24 patients) once the guide catheter and guide wire were in position prior to the PCI procedure. Balloon inflations of between 30 s and 1 min were performed to predilate the target lesions. Serial venous samples were then collected from either the coronary sinus or femoral vein at 1 and 5 min post-balloon inflation. PCI was then completed as per routine at our center. Twelve hour post-procedure samples to measure sphingolipid and troponin T levels were taken from a peripheral vein.

After PCI, blood samples were immediately dispensed into 3 ml ethylenediaminetetraacetic acid (EDTA) tubes with 2-chloroadenosine (0.05 mmol/liter) and procaine hydrochloride (0.154 mol/liter) and equilibrated at 4°C. All blood samples collected were centrifuged briefly to clarify and kept at 4°C. Once samples are derivatized, they were diluted into the mobile phase, kept at 0–4°C, and analyzed by HPLC as soon as possible. As an added precaution, standards were alternated with samples to detect (and correct for) losses over time.

Only patients with angiographic single vessel disease undergoing elective PCI participated in this study, and had documented normal left ventricular and renal function. Patients with history of coronary artery bypass graft (CABG), valvular heart disease, or MI/acute coronary syndrome (ACS) were excluded as were PCI procedures in patients with chronic total occlusions. Peripheral blood samples were also taken from 11 healthy controls with no history of CAD.

### High-performance liquid chromatography

S1P standards were purchased from Avanti Polar Lipids, Inc. (Delfzyl, The Netherlands). All other chemicals, including *o*-phthaldialdehyde (OPA), D-sphingosine, D-erythro-dihydrosphingosine, boric Acid, β-mercaptoethanol were purchased from Sigma-Aldrich (Dorset, UK). All solvents for high-performance liquid chromatography (HPLC) were purchased from Fisher Scientific (Leicestershire, UK). All blood samples collected into EDTA with 2-chloroadenosine and procaine during the procedure were centrifuged at 2056 g for 15 min at 4°C. Aliquots of plasma (0.5 ml) were stored at −80°C until analyzed by HPLC. Sphingolipids were extracted from samples and HPLC analysis of sphingolipids (S1P, SPH, and SA) levels were performed according to Caligan et al. (2000).

### Determination of troponin T and high sensitive troponin T (hsTnT)

Troponin T level in peripheral vein at 12 h after post-PCI was measured by standard assay (Roche Troponin T). The level of high sensitive troponin T (hsTnT) in samples from both the coronary sinus and femoral vein at 1 and 5 min after post-PCI was also measured by High-Sensitive Troponin T assay (Roche Diagnostics; Helleskov Madsen et al., 2008). The lower detection limits of standard and hsTnT assays are 0.01 ug/l and 5 ng/l, respectively.

### Determination of Ox-LDL and hsCRP

Ox-LDL was measured by a commercially available sandwich ELISA (Mercodia) with specific monoclonal antibody mAb-4E6 as described by Holvoet et al. (1998). A standard curve showing the binding range of Ox-LDL samples was prepared. Internal controls consisting of high and low standard plasma samples were included on each microtiter plate to detect potential variations between microtitration plates. Each sample was assayed in triplicate. The intra-assay coefficients of variation for all assays were 5–9%. High resolution CRP (hsCRP) was measured using a high-sensitivity assay with reagents and a BNII analyzer from Dade-Behring, Milton Keynes, UK. The intra- and inter-assay coefficient of variation for the hsCRP assay was 3.9 and 4.6%, respectively.

### Statistical analysis

All data are reported as means ± sem. Repeated measure One-Way ANOVA was used to compare values of measurements between groups. When analysis of variance revealed a significant difference among values, Tukey's test was applied to determine the significance of a difference between selected group means. *P* < 0.05 was taken to indicate statistical significance.

## Results

Blood samples were taken from total of 31 study participants undergoing routine elective PCI. Characteristics and coronary lesion data of the study cohort are presented in Table 1. Pre-dilation of the target lesions was performed with angioplasty balloons inflated between 14 and 22 Atmospheres (mean 15 Atmospheres) for a period of between 28 and 40 s (mean 31.1 s). During balloon inflation ischemic ECG changes were noted in 20/31 patients (64.5%) with ST elevation in 13/31 patients (41.9%, mean ST elevation 0.5 mm) and ST depression in 7/31 patients (22.5%, mean ST depression 0.5 mm). In the remaining 11/31 patients (35.4%) no ECG changes were observed although all patients reported transient chest discomfort during this period.

**Table 1:** **Characteristics and coronary lesion data of the study cohort (*n* = 31)**.

**DEMOGRAPHICS**	
Age (Years; mean ± SEM)	63 ± 9
Sex (% Male)	97
Caucasian (%)	87
% Normal LV function (EF > 60%)	100
% Normal renal function	100
**RISK FACTORS**	
Hypertension (%)	53
Diabetes (%)	15
Hyperlipidemia (%)	85
Smoking (%)	39
BMI (kg/m^2^)	27.4 ± 4.8
**MEDICATION**	
Antiplatelet therapy (%)	100
B-Blockers (%)	82
ACEi (%)	65
Statins (%)	100
Nitrates (%)	22.5
Ca Blockers (%)	29.5
**TARGET VESSEL**	
LAD	21/37 (67.7%)
RCA	8/31 (25.8%)
Cx	2/31 (6.4%)
**VESSEL DIAMETER**	
2.5–2.99 mm	12/31 (38.7%)
3–3.49 mm	11/31 (35.4%)
3.5–3.99 mm	7/31 (22.5%)
4.5–4.99 mm	1/31 (3.2%)
**LESION LENGTH**	
10–14 mm	4/31 (12.9%)
15–19 mm	5/31 (16.1%)
20–24 mm	11/31 (35.4%)
25–30 mm	8/31 (25.8%)
>30 mm	8/31 (25.8%)
**% STENOSIS**	
50–74%	2/31 (6.4%)
75–94%	22/31 (70.9%)
>95%	7/31 (22.5%)

**Figure 1:**
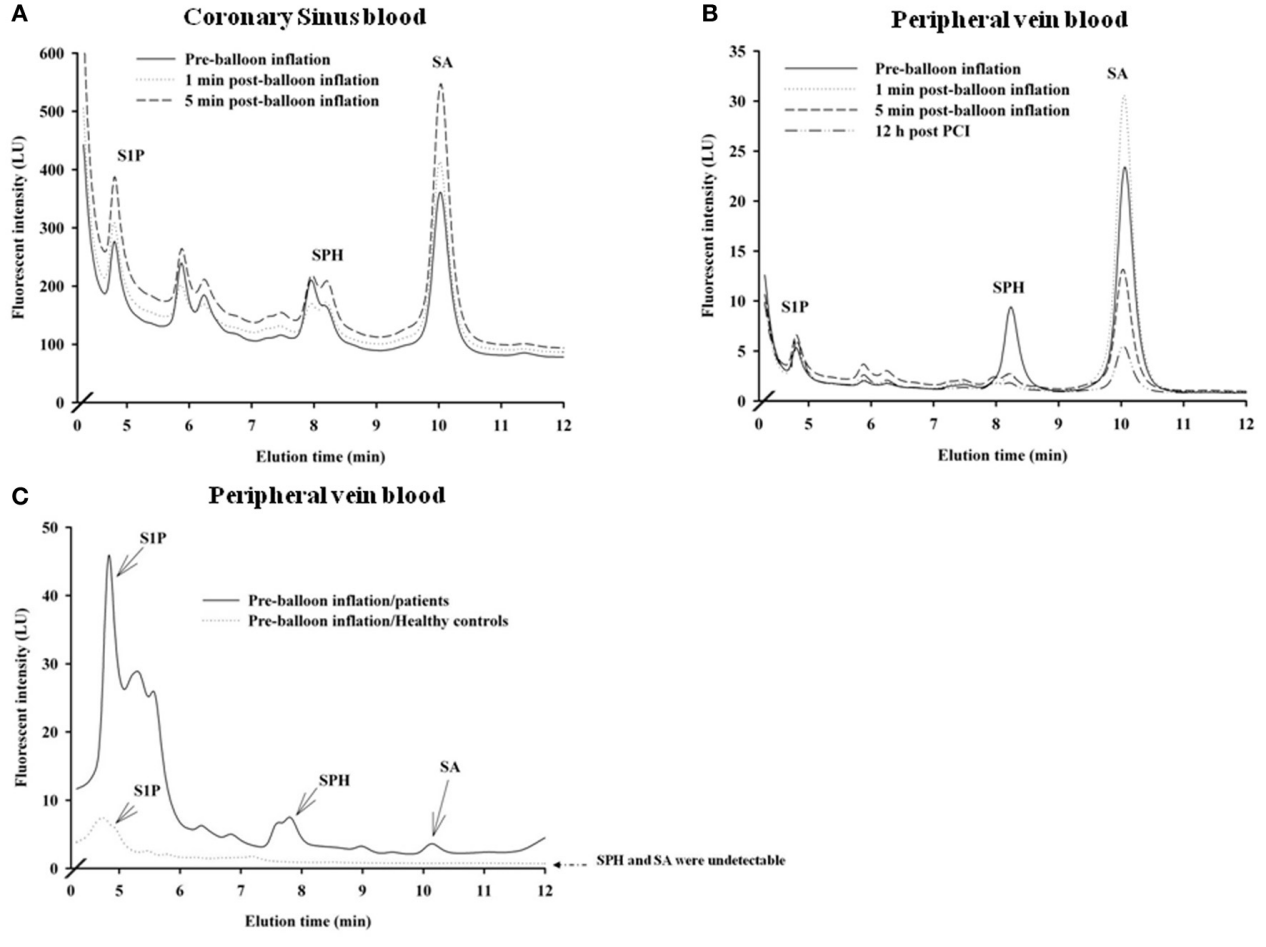
**Distribution of sphingolipids in coronary sinus blood (A; *n* = 7) and in peripheral blood (B; *n* = 24) in patients who underwent PCI**. Also shown are comparative distributions of sphingolipids in peripheral blood at baseline (before balloon inflation in a patient) and in a blood sample from a healthy control (**C**; *n* = 11). Representative examples of relative plasma levels of sphingolipids as detected by HPLC at baseline and at different time points: 1, 5 min, and 12 h post-PCI are shown.

**Figure 2:**
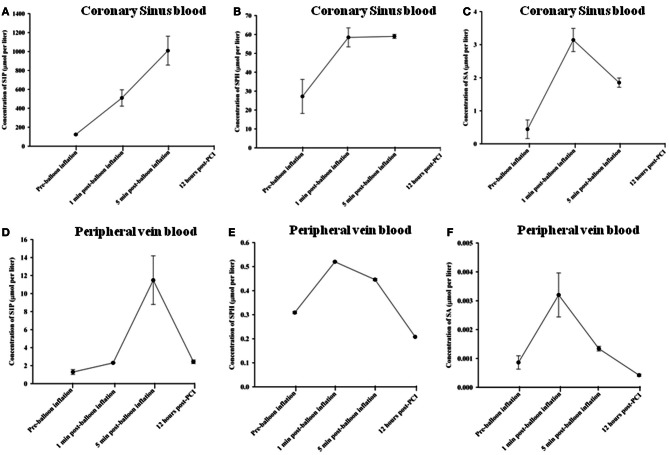
**Changes in sphingolipid concentrations in coronary sinus blood: S1P (A); SPH (B); and SA (C) and comparative concentrations in peripheral blood for S1P (D); SPH (E); and SA (F), at different time course points following balloon inflation**.

Using HPLC we analysed plasma levels of sphingolipids in patients at baseline (pre-balloon inflation) and at different time course points after balloon inflation (1, 5 min, and 12 h). HPLC analysis revealed significant alterations in plasma levels of sphingolipids sampled from the coronary sinus from 7 patients and peripheral veins from 24 patients following induction of transient myocardial ischaemia by balloon occlusion of target lesion. Representative examples of isolation and detection of sphingolipids, at baseline and at different time course points after balloon inflation are shown in Figures 1A,B. Baseline concentrations of S1P measured from peripheral blood samples were more than 4-fold higher in patients with documented CAD undergoing PCI compared to healthy controls (1.29 ± 0.27 vs. 0.38 ± 0.05 μmol/liter; *n* = 11; *P* < 0.001; Figure 1C). As illustrated in Table 2, there was a significant increase in all three sphingolipid levels at 1 and 5 min, compared with baseline levels, both in coronary sinus blood (Figures 2A,B,C) and peripheral blood (Figures 2D,E,F). S1P showed the largest increase of the three sphingolipids, with its greatest level at 5 min (Figures 2A,D), whereas the levels of SPH and SA were highest at 1 min and began to decrease at 5 min (Figures 2B,C,E,F).

**Table 2:** **Plasma levels of sphingolipids in coronary sinus and peripheral blood in patients who underwent PCI**.

**Time**	**Coronary sinus (μmol per liter) ***n*** = **7****			**Peripheral (μmol per liter) ***n*** = **24****		
	**S1P**	**SPH**	**SA**	**S1P**	**SPH**	**SA**
Baseline	123.34 ± 7	27.19 ± 9	0.44 ± 0.28	1.23 ± 0.27^†^	0.31 ± 0.004^†^	0.0009 ± 0.0002^†^
1 min	509.13 ± 86^*^	58.45 ± 5^*^	3.14 ± 0.35^*^	2.31 ± 0.06^*^^†^	0.52 ± 0.002^*^^†^	0.0032 ± 0.001^*^^†^
5 min	1008.8 ± 152^*^	59 ± 1.08^*^	1.85 ± 0.14^*^	11.48 ± 2.70^*^^†^	0.45 ± 0.005^*^^†^	0.0013 ± 0.0001^*^^†^
12 h	N/A	N/A	N/A	2.42 ± 0.20^*^	0.21 ± 0.001	0.0004 ± 0.00004

Concentrations are shown for baseline (pre-balloon inflation) and at 1 and 5 min post-inflation both for coronary sinus and peripheral and also 12 h post-PCI for peripheral samples. Values are expressed as mean ± sem. N/A denotes not available.

* P < *0.001* for the comparison between the baseline vs. 1 and 5 min post-balloon inflation.

† P < *0.001* for comparison between coronary sinus and peripheral levels.

At 1 min following balloon inflation, in coronary sinus, levels of S1P, SPH, and SA increased by 314, 115 and 614%, respectively, compared with baseline levels (*n* = 7, all *P* < 0.001), whereas in peripheral blood, levels of S1P, SPH, and SA increased by 79, 68, and 272%, respectively, compared with baseline levels (*n* = 24, all *P* < 0.001). Peripheral sphingolipid levels at 1 min were consistently very much lower than coronary sinus levels. At 5 min after balloon inflation, in coronary sinus blood, levels of S1P, SPH, and SA increased by 720, 117, and 320% compared with baseline levels (*n* = 7, all *P* < 0.001), while in peripheral blood, levels of S1P, SPH, and SA increased by 792, 44, and 56% compared with baseline levels (*n* = 24, all *P* < 0.001). At 12 h following the PCI procedure, peripheral levels of S1P were much lower than that at 1 or 5 min, but were still elevated compared to baseline [S1P: by 88% (*n* = 24, all *P* < 0.001)]. Peripheral SPH and SA levels had declined to below baseline (decrease of SPH: 33% *n* = 24, all *P* < 0.001; SA: 51%, *n* = 24, all *P* < 0.001).

**Figure 3:**
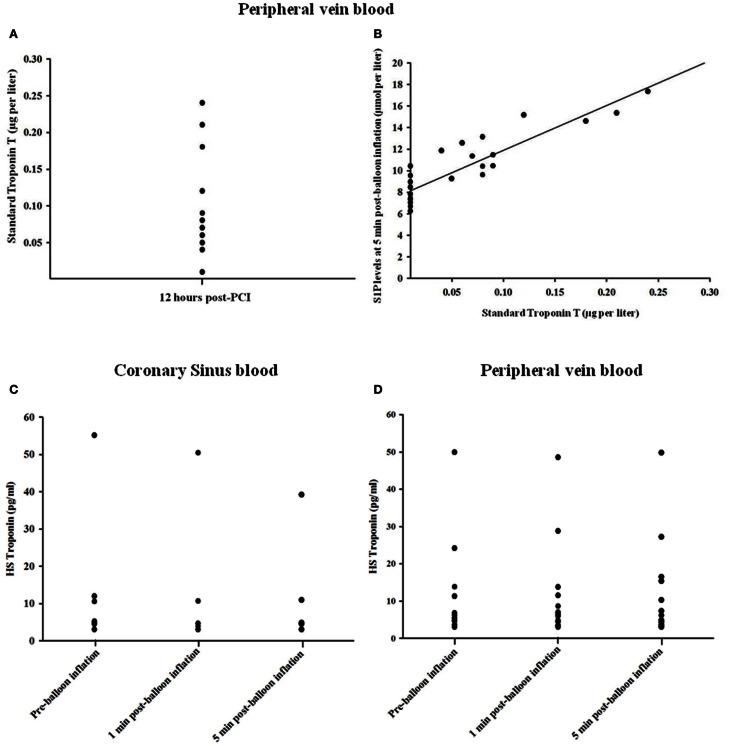
**Relationship between troponin T levels at 12 h (A) and peak S1P levels (B) in the study participants**. In those cases where troponin T was detectable there was close correlation between troponin T and S1P levels (*R*^2^ = 0.818; *P* < 0.0001). **(C,D)** Changes in hsTnT concentration in coronary sinus blood and peripheral blood.

To determine whether the observed increase in sphingolipids following transient coronary occlusion was related to myocardial necrosis or cardiac ischaemia *per se*, serum troponin levels were measured. Elevated 12 h troponin T levels were detectable in only 10 of 31 study subjects (32.3%), following PCI (Figure 3A), whereas S1P concentrations were elevated in all subjects studied. Figure 3B shows 12 h troponin T levels plotted against peak S1P level in the study participants. For those individuals in whom troponin T was detectable at 12 h, a strong correlation was found between peak serum SIP levels and 12 h troponin T level (*R*^2^ = 0.818; *P* < 0.0001). Furthermore no significant changes occurred in hsTnT levels in either the coronary sinus (*n* = 6) or peripheral blood (*n* = 12) at 1 and 5 min time points after transient coronary occlusion as shown in Figures 3C,D, respectively.

**Figure 4:**
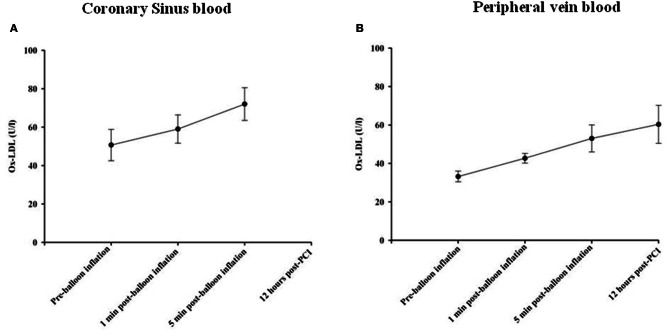
**Changes in Ox-LDL concentrations in coronary sinus (A) and peripheral (B) blood at different time course points following balloon inflation**.

Oxidized LDL (OxLDL) was measured as a biomarker of oxidative stress in the serum samples obtained. OxLDL levels in coronary sinus and peripheral blood at different time points are shown in Figures 4A,B, respectively. At 1 min following balloon inflation, in coronary sinus, levels of OxLDL increased by 16%, compared with baseline levels (*n* = 7, *P* = 0.29), whereas in peripheral blood, levels increased by 29% compared with baseline (*n* = 24, all *P* < 0.001). Peripheral OxLDL levels were consistently lower than coronary sinus levels. At 5 min after balloon inflation, in coronary sinus blood, levels of OxLDL increased by 42% compared with baseline (*n* = 7, all *P* < 0.001), while in peripheral blood, levels of OxLDL increased by 60% compared with baseline (*n* = 24, all *P* < 0.001). At 12 h following the PCI procedure, levels of OxLDL increased by 82% compared with baseline (*n* = 7, all *P* < 0.001 95% CI).

**Figure 5:**
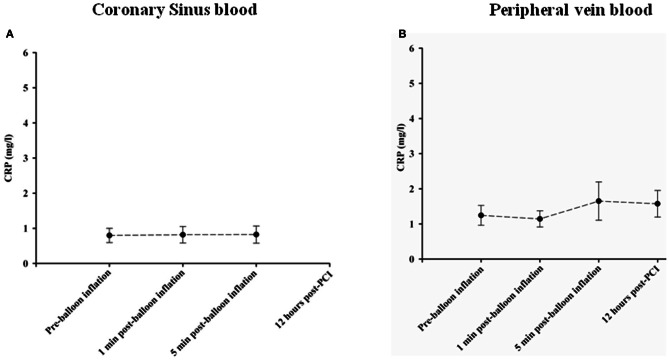
**Changes in CPR concentrations in Coronary sinus (A) and peripheral (B) blood at different time course points following balloon inflation**.

hsCRP levels were measured as a general marker of inflammation. No significant changes occurred in hsCRP levels in coronary sinus and peripheral blood at different time points as shown in Figures 5A,B, respectively.

## Discussion

This study demonstrated for the first time plasma sphingolipid behavior following transient cardiac ischaemia in humans, with dramatic increases in S1P, SPH, and SA. Levels were markedly increased in both the coronary sinus and peripherally within 1 min of transient ischaemia mediated by short periods of coronary vessel occlusion. To date, no biomarker used in clinical practice has the ability to detect such transient episodes of cardiac ischaemia as we observed with sphingolipids.

Cardiac biomarkers, such as cardiac troponins, have become the standard test in combination with clinical and electrocardiographic findings to diagnose and risk stratify patients with ACS. Recently two studies reported the early diagnosis of MI with high sensitive troponin assays, demonstrating this to be more accurate in diagnosing MI, compared to the conventional troponin test and other markers (Keller et al., 2009). These high sensitivity tropnin assays have high diagnostic specificity and sensitivity for the diagnosis of ACSs after only 2 or 3 h following the onset of chest pain (Keller et al., 2009; Bonaca et al., 2010). The enhancements in troponin assays have enabled resolution of the 99th percentile reference limit at progressively lower concentrations. However, the clinical significance of low-level increases with sensitive assays is still debated (Bonaca et al., 2010).

Plasma S1P, SPH, and SA levels are more sensitive markers of transient cardiac ischaemia in the subjects studied in this report compared to either hsTnT or regular TnT since we did not observe a significant elevation of hsTnT in coronary samples of the subjects at 1 and 5 mins following PCI despite marked changes in sphingolipid levels. Indeed, previous studies have shown that the greatest diagnostic utility of hsTnT is between 2 and 6 h following ischemic insult (Keller et al., 2009; Bonaca et al., 2010) which is several orders of magnitude longer than we have observed with our sphingolipid markers. Furthermore, elevated troponin T level was detectable at 12 h following PCI in 10 of 31 study subjects (32.3%).

Significant changes observed in oxidized LDL, a biomarker of oxidative stress, over the same time-course as the changes in sphingolipids may suggest a role for oxidative stress in the regulation of sphingolipid metabolism as described in animal studies (Maceyka et al., 2007). The peripheral baseline ox-LDL levels in our study are similar to those reported by Holvoet et al. (1998) in their comparison of ox-LDL levels between healthy controls and CAD patients. Tsimikas and co-workers found, in patients presenting at the Emergency Room with chest pain, that circulating ox-LDL specific markers strongly reflect the presence of ACS (Holvoet et al., 2006). Ehara et al. showed that plasma ox-LDL levels were significantly higher in AMI patients than in stable angina patients (Ehara et al., 2008) Interestingly, the magnitude of increase of ox-LDL observed in our study was much greater than those observed in AMI patients in Ehara et al.'s study suggesting that such an increase would be unlikely due to a rupture of unstable plaques. Buffon et al. (2000) have similarly observed transient (<15 min' duration) elevation of free lipid peroxides in the coronary sinus during balloon occlusion of the left anterior descending coronary artery. It is possible that ROS generated secondary to such ischemic insults may oxidize phospholipids in the vessel wall or even in plasma, which would then be subsequently detected as OxLDL in plasma. It is well-known that during the onset of hypoxia, ROS activates neutral sphingomyelinase, generating ceramide. Furthermore, ROS also leads to activation of Sphingosine Kinase 1 in a PKC-dependent manner (Jin et al., 2002), hence some of this ceramide may be metabolized to S1P thereby increasing S1P levels, in line with our observations. Indeed, the differences in sphingolipid response observed during cardiac ischaemia between our study and that of Knapp et al. (2009) in which they observed a decrease in S1P levels following MI may be due to the more prolonged ischaemia during MI resulting in degradation of sphingosine kinase 1 (Maceyka et al., 2007) and an inhibition of its activity mediated by ROS leading to decreased levels of S1P and its metabolites (Knapp et al., 2009).

Distinct changes in levels of S1P, SPH, and SA over the 12 h time course of sampling reflect the complex dynamic metabolism or inter-conversion of these three sphingolipids in the coronary and peripheral circulation. Yatomi et al. (1995) showed that about 50% of SPH was converted to S1P within 5 min in intact platelets and plasma. These results may explain why our SPH plasma levels tend to decrease and S1P increases after 5 min. S1P has been found to be metabolically stable for at least 2 h (Yatomi et al., 1995) so once released from cardiac myocytes is likely to circulate in the body, which could account for the 11% of the 5 min S1P peripheral blood levels that was still detectable at 12 h. Consistently, Sattler et al showed that S1P levels in plasma rise during the first 12 h of MI and decline thereafter. The authors demonstrated that in this setting plasma high density lipoprotein-C (HDL-C), but not other carriers, is the acceptor of S1P as mirrored by the increase in their S1P content to concentrations exceeding even those of healthy HDL (Sattler et al., 2010). The decrease in levels of SA may also be attributed to its very fast turnover (Yatomi et al., 1997).

Our study also indicates a potential role of sphingolipids in pathophysiological processes that occur during early cardiac ischaemia. Vessey and colleagues have demonstrated that sphingolipids are important endogenous cardioprotectants released by ischemic pre- and post-conditioning in experimental animal models (Vessey et al., 2009) and pre-treatment with exogenous S1P provides protection against cardiac I/R injury (Karliner, 2009). Kelly et al. have shown that ethanolamine, a metabolite of S1P protects the murine heart against I/R injury via activation of STAT-3 (Kelly et al., 2010). In addition, Theilmeier et al. have shown that the HDL and its constituent, S1P, acutely protect the heart against ischaemia/reperfusion injury *in vivo* via an S1P3-mediated and nitric oxide-dependent pathway, suggesting that a rapid therapeutic elevation of S1P-containing HDL plasma levels may be beneficial in patients at high risk of acute myocardial ischaemia (Theilmeier et al., 2006). Interestingly, Sattler et al. have recently shown that the amount of plasma S1P not bound to HDL and the ratio of non-HDL-bound and HDL-bound S1P in plasma are increased in patients with stable CAD and MI compared to healthy individuals and are correlated to the clinical severity of CAD symptoms (Sattler et al., 2010). In addition the authors also found that the amount of plasma S1P not bound to HDL is inversely associated with the S1P content of isolated HDL only in healthy individuals but not in patients with CAD, implying a functional alteration in the S1P exchange between HDL-bound and non-HDL-bound S1P plasma pools in CAD. We recently found that the S1P receptor agonist, FTY720, a new generation of S1P receptor modulator in phase III clinical trials as an immuno-suppressant agent for the treatment of auto-immune diseases and in organ transplantation (Budde et al., 2006), can prevent ischaemia-reperfusion damage in isolated heart and sino-atrial (SA) nodes in the rat (Egom et al., 2010a). We also showed that FTY720 reduces ischaemia-induced ventricular arrhythmias and SA nodal dysfunction via activation of p21-activated kinase (Pak1), a Ser/Thr kinase downstream of small G-proteins, and Akt (Egom et al., 2010a,b). FTY720 may also inhibit atherosclerosis by suppressing the machinery involved in monocyte/macrophage emigration to atherosclerotic lesions (Keul et al., 2007). In contrast to S1P receptors on lymphocytes, the authors also demonstrated that FTY720 did not desensitize vascular S1P receptors suggesting that S1P agonists that selectively target the vasculature and not the immune system may be promising new drugs against atherosclerosis (Keul et al., 2007). In addition, S1P_3_ receptor may mediate the chemotactic effect of S1P in macrophages *in vitro* and *in vivo* and may play a causal role in atherosclerosis by promoting inflammatory monocyte/macrophage recruitment and altering smooth muscle cell behavior (Keul et al., 2011).

Recently, several novel biomarkers including hFABP (Colli et al., 2007) and cMyBP-C (Govindan et al., 2012) have been proposed. hFABP is a small soluble cytosolic protein involved in the transportation of long-chain fatty acids into the cardiomyocyte is released rapidly into the circulation in response to cardiomyocyte injury. Due to its solubility, hFABP can be released more rapidly than structurally bound molecules like cardiac troponins. Furthermore, it may enter the vascular system directly via endothelium because of its small size (15 kDA). Thus, hFABP is regarded as an early sensitive marker of AMI (Colli et al., 2007). Cardiac myosin binding protein-C (cMyBP-C) is a thick filament assembly protein that stabilizes sarcomeric structure and regulates cardiac function; however, the profile of cMyBP-C degradation after MI is unknown (Govindan et al., 2012).

Are sphingolipids mechanistically relevant to ischaemia-related arrhythmias? We have recently demonstrated that the S1P agonist FTY720 may reduce ischaemia-induced ventricular arrhythmias and SA nodal dysfunction via activation of Pak1, a Ser/Thr kinase downstream of small G-proteins, and Akt (Egom et al., 2010a). The detailed mechanisms underlying this protective effect is likely to be complex, and may involve primary effects on ion channels/transporters and secondary effect to protect cardiac myocytes from hypoxia-or ischemia-induced stress and cell death. Both S1P and FTY720 may have functional effect through I_K,ACh_ as suggested by previous studies (Guo et al., 1999; Koyrakh et al., 2005).

The current study has number of limitations. Firstly, the study was conducted in a limited number of patients hence larger studies are needed to validate the value of serum sphingolipids level in the early detection of cardiac ischaemia. Secondly, transient balloon dilatation is a very different process from plaque rupture and thrombus/emboli occlusion in clinical situation. Therefore, the clinical implication of the findings in this study need to be further studied in larger number of cases of ACSs. Thirdly, since this was a prospective, observational study, we cannot quantify the clinical effect associated with the increase in early diagnostic accuracy. Fourthly, the specificity of serum sphingolipids in the early detection of cardiac ischaemia is uncertain, since it is possible that sphingolipids may also be elevated in other cardiovascular diseases as well as in situations such as sepsis and a variety of other inflammatory processes that evoke the release of inflammatory cytokines and involve the TNF pathway. However, hsTnT have also been shown to be elevated in aortic dissection, valvular heart disease and acute decompensated heart failure (Keller et al., 2009). Finally, it is still to be decided whether the elevation in Sphingolipids from these patients is derived from vascular trauma due to PCI or from myocardial ischaemia and/or necrosis, although the strong correlation with 12 h troponin levels would suggest that they are less likely to be derived from vascular trauma alone. In addition, there is no significant correlation between ECG changes (ischaemia phenotype) and plasma levels of S1P, SPH, and SA, This may be due to a limited number of patients involved in this cohort. However, even in those patients who did not have ECG changes, ischemic symptoms were experienced.

In conclusion, we demonstrate the behavior of plasma sphingolipids level in transient cardiac ischaemia in humans produced by transient coronary vessel occlusion observed during PCI. The results show a dramatic increase in plasma S1P, SPH, and SA levels at very early stages of ischaemia, correlating strongly with Troponin levels 12 h post-PCI. These molecules therefore may become novel potentially robust early predictors of acute myocardial ischaemia presenting with ACS therefore provide a crucial timing window for treating this condition for preventing the occurrence of fatal ventricular arrhythmias. This study also raises the question of whether modulating the sphingolipid pathway may lead to potential therapeutic benefit both before and during an ischemic coronary event.

## Funding sources

This research was supported by the Wellcome Trust, MRC, and BHF (Ming Lei), Emmanuel E. Egom is a recipient of the Heart and Stroke Foundation of Canada Fellowship, British Heart Foundation (Ming Lei, Sally E. Stringer), The Biomedical Research Centre (Mamas A. Mamas, Ludwig Neyses, Farzin Fath-Ordoubadi). MHC coronary intervention research fund (Farzin Fath-Ordoubadi).

### Conflict of interest statement

The authors declare that the research was conducted in the absence of any commercial or financial relationships that could be construed as a potential conflict of interest.
